# Skills-based intervention to enhance collaborative decision-making: systematic adaptation and open trial protocol for veterans with psychosis

**DOI:** 10.1186/s40814-021-00820-4

**Published:** 2021-03-29

**Authors:** Emily B. H. Treichler, Borsika A. Rabin, William D. Spaulding, Michael L. Thomas, Michelle P. Salyers, Eric L. Granholm, Amy N. Cohen, Gregory A. Light

**Affiliations:** 1 VA Desert Pacific Mental Illness Research, Education, and Clinical Center (MIRECC), San Diego, CA USA; 2 Department of Psychiatry, University of California San Diego, 9500 Gilman Drive 0804, La Jolla, CA 92093 USA; 3 Department of Family Medicine and Public Health, University of California San Diego, La Jolla, CA USA; 4 Center of Excellence in Stress and Mental Health, San Diego VA, La Jolla, CA USA; 5 UC San Diego Dissemination and Implementation Science Center, University of California San Diego, La Jolla, CA USA; 6 Department of Psychology, University of Nebraska-Lincoln, Lincoln, NE USA; 7 Department of Psychology, Colorado State University, Fort Collins, CO USA; 8 Department of Psychology, Indiana University-Purdue University at Indianapolis, Indianapolis, IN USA; 9 VA San Diego Psychology Service, San Diego, CA USA; 10 American Psychiatric Association, Los Angeles, CA USA; 11 Department of Psychiatry and Biobehavioral Sciences, University of California Los Angeles, Los Angeles, CA USA

**Keywords:** Shared decision-making, Patient activation, Schizophrenia, Implementation science, Pilot, Recovery model, Mixed methods, Adaptation, Participatory methods

## Abstract

**Background:**

Collaborative decision-making is an innovative decision-making approach that assigns equal power and responsibility to patients and providers. Most veterans with serious mental illnesses like schizophrenia want a greater role in treatment decisions, but there are no interventions targeted for this population. A skills-based intervention is promising because it is well-aligned with the recovery model, uses similar mechanisms as other evidence-based interventions in this population, and generalizes across decisional contexts while empowering veterans to decide when to initiate collaborative decision-making. Collaborative Decision Skills Training (CDST) was developed in a civilian serious mental illness sample and may fill this gap but needs to undergo a systematic adaptation process to ensure fit for veterans.

**Methods:**

In aim 1, the IM Adapt systematic process will be used to adapt CDST for veterans with serious mental illness. Veterans and Veteran’s Affairs (VA) staff will join an Adaptation Resource Team and complete qualitative interviews to identify how elements of CDST or service delivery may need to be adapted to optimize its effectiveness or viability for veterans and the VA context. During aim 2, an open trial will be conducted with veterans in a VA Psychosocial Rehabilitation and Recovery Center (PRRC) to assess additional adaptations, feasibility, and initial evidence of effectiveness.

**Discussion:**

This study will be the first to evaluate a collaborative decision-making intervention among veterans with serious mental illness. It will also contribute to the field’s understanding of perceptions of collaborative decision-making among veterans with serious mental illness and VA clinicians, and result in a service delivery manual that may be used to understand adaptation needs generally in VA PRRCs.

**Trial registration:**

ClinicalTrials.gov Identifier: NCT04324944

## Contributions to the literature

- Collaborative decision-making is desired by veterans with serious mental illness, but current rates are low. This study is the first to adapt and evaluate a collaborative decision-making intervention, a brief group skills training focused on empowerment, concrete knowledge and skills, and communication.
- This study will improve understanding about how to work with a stakeholder mental health team including both patients and providers to systematically adapt and evaluate a new intervention.
- Lessons learned from this study will be disseminated to similar mental health clinics to support evidence-based adaptation and increase collaborative decision-making between patients and providers.

## Background

Recovery-oriented care is a holistic, self-directed, and empowering process that emphasizes personal meaning and quality of life in care choices, as opposed to a traditional approach that prioritizes symptom remission specifically [1]. This model continues to monitor and intervene on symptomatic outcomes but focuses on personally prioritized functional outcomes and wellbeing. One outcome of recovery-oriented services is enhanced sense of personal recovery, a patient’s endorsement of hope, optimism, social connectedness, personal meaning, and empowerment [2].This model of care is a particularly good fit for veterans with serious mental illnesses, given high rates of long-term functional disability and low rates of full symptom remission [3, 4]. The Interdepartmental Serious Mental Illness Coordinating Committee, a federal committee intended to improve care for people with SMI, prioritizes using recovery-oriented care to optimize quality of care and improve functional outcomes [5]. Changes in rehabilitation programming for veterans with SMI are beginning to reflect this priority, for example by integrating peer services into Psychosocial Rehabilitation and Recovery Centers (PRRCs). However, much work is needed to effectively develop and implement recovery-oriented care for veterans with SMI in the Veteran’s Affairs (VA) system [6]. A key gap in current recovery-oriented care is interventions and other processes that facilitate empowering and person-centered processes within the treatment process itself, including effective interaction with providers and meaningful involvement in treatment decision-making.

Recovery-oriented care includes an emphasis on veteran self-direction within the treatment process, as well as facilitating veteran empowerment via intervention approaches. The recovery model posits that the treatment process itself can lead to improved sense of personal recovery if the process is empowering and facilitates hope [7]. Studies of people with SMI, including veterans, indicate that patient involvement in treatment decision-making is associated with a range of positive outcomes, including improved treatment engagement, treatment satisfaction, psychological functioning, social functioning, and quality of life [7–10]. On the other hand, lack of patient involvement in treatment decisions is associated with increased risk of attrition and lower treatment adherence [11, 12].

Interest in involvement in treatment decision-making among this population is high. Eighty-five percent of veterans with SMI prefer to be involved in treatment decision-making, including having options to choose from and being asked their opinion by providers [13]. However, studies across relevant populations have found that rates of patient involvement in treatment decision-making are low [14, 15]. For example, one study found that during medication appointments between psychiatrists and patients with SMI, the final decision only reflected patient preferences 22% of the time, including decisions where psychiatrist and patient preference were the same [15].

### Rationale for collaborative decision-making

Patient involvement in treatment decision-making is most typically called *shared decision-making* [16]. We argue for a recalibration of the shared decision-making concept to *collaborative decision-making* [17]. Providers believe they are engaging in “shared” decision-making when they are communicating something to a patient, i.e., a diagnosis, potential side effects of a medication, and make decisions about whether to “share” decision-making with patients based on patient characteristics like illness severity [18–20]. The new collaborative decision-making model places equal power and responsibility over the decision with each person involved. It seeks to empower the patient during the process, in keeping with the recovery model. “Collaboration” makes it clear that this model is about a reciprocal exchange of ideas, a process where both the patient and provider (and potentially, other stakeholders) have a meaningful say. The collaboration decision-making process prioritizes the patient’s needs, values, and culture along with best evidence and clinical judgment by actively integrating these through collaboration, actualizing the evidence-based practice model.

### Existing relevant interventions

A range of interventions targeting shared decision-making have been developed, although none have been developed specifically for veterans with SMI. These interventions are most frequently decision support tools or decision aids, which are provider- or clinic-based interventions intended to facilitate decision-making around specific decisions [21]. Some of these interventions are effective in improving treatment engagement and satisfaction, social functioning, quality of life, and clinical outcomes [21–28]. However, there are limitations to these approaches. First, these types of interventions target a single decision, limiting their reach. Second, these interventions rely on the provider or clinic for access or initiation with each decision-making process, undermining patient empowerment. This is especially concerning because even providers who endorse shared decision-making are less likely to use shared decision-making principles if they view the patient as too symptomatic [19]. In general, physicians rarely initiate shared decision-making processes but often react positively to patient initiation of shared decision-making [29, 30]. When patients initiate the decision-making process, treatment decisions are more likely to reflect patient preference [15]. Finally, while some types of decisions are well-suited to a decision aid (i.e., when there are two or three concrete, easy to distinguish choices), many treatment decisions are more complicated and options may vary widely by individual, and therefore less well-suited to be resolved using a decision aid (e.g., where to live, who to choose as a guardian or fiduciary). Decision contexts also vary, particularly for people with SMI, who often have a range of providers and other people (e.g., family members) contributing to treatment and treatment decision-making. For example, in VA Psychosocial Rehabilitation and Recovery Centers (PRRCs), veterans with SMI have interdisciplinary teams which may include a psychiatrist or other prescriber, an individual therapist, a recovery coach, group therapists, a vocational therapist, a recreational therapist, a peer support specialist, and a chaplain. An innovative and comprehensive approach, CommonGround, has shown promise for adults with SMI, but barriers at multiple levels obstruct successful implementation [31, 32]. Combined with high rates of turnover in many clinical settings, targeting veterans rather than providers may be a more feasible and sustainable approach.

These issues indicate that an intervention that focuses on empowering patients and providing generalizable decision-making skills may be an effective method for a wide range of decisions and decision contexts. This type of approach has previously been found beneficial for adults with more mild to moderate mental health issues [33]. Further, supporting patient initiation of collaborative decision-making is congruent with the recovery model, given the emphasis on autonomy and empowerment. Given past studies [28] indicating increased patient-provider collaboration may be effective because of improved self-management and self-efficacy, the focus on autonomy and empowerment is also congruent with understandings of how to increase treatment collaboration in an evidence-based manner.

### Collaborative Decision Skills Training (CDST)

**Fig. 1:**
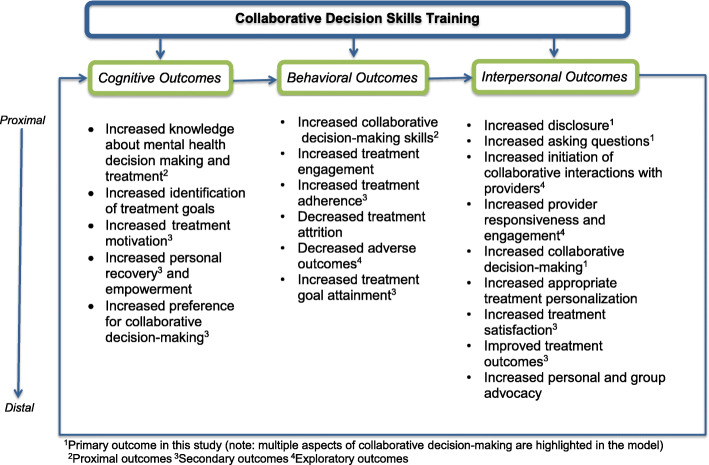
CDST theoretical model

Collaborative Decision Skills Training (CDST) was developed in pursuit of increasing patient initiation and engagement in collaborative decision-making, as well as filling a gap in available collaborative decision-making tools for patients with SMI [34]. E.T. conceptualized and wrote the manual with mentorship from W.S. and Eric A. Evans. The theoretical model of CDST and its outcomes are depicted in Fig. 1. A skills training intervention was chosen because patient initiation of collaborative decision-making usually results in a positive response from providers, indicating that training patients in skills needed to initiate these interactions is a viable strategy. Additionally, other skills training interventions are effective and generalizable to functional outcomes among people with SMI [35]. Finally, skills training is a particularly feasible option due to its ease of implementation, e.g., requiring few financial or technical resources, building on existing provider competencies. Skills training may increase the effectiveness of existing provider-based tools, like decision aids, because some patients may not currently be able to fully utilize these tools due to lack of knowledge, skill, or confidence in engaging in collaborative decision-making [36]. This approach also has the promise to expand use of collaborative decision-making across types of decisions and providers. CDST is intended to enable patients to initiate collaborative decision-making with any treatment team member regarding any decision and define their own preferred role in decision-making, unlike most SDM interventions, which target specific decisions or providers. Therefore, the primary targeted outcome of CDST is increased collaborative decision-making behaviors, a valued outcome in itself by stakeholders, and also associated with downstream outcomes including improved treatment engagement and satisfaction, better treatment outcomes, and better quality of life.

CDST is a solution-focused skills training group covering five primary areas: psychoeducation about treatment decision-making processes, empowerment, assertiveness skills, problem-solving skills, and conflict management skills. Participants attend CDST in small groups of four to ten people, for eight 1-h sessions, with homework between sessions. Participants identify their treatment teams, their preferred roles in treatment decisions, current rehabilitation goals, and any rehabilitation-related concerns or obstacles. Participants practice skills to help them participate in treatment decision-making and to make independent decisions about treatment-related goals and concerns. Special topics are introduced, including handling conflict with providers and advocating for system-level change. Its fundamental components or “active ingredients” are listed in Table 1.

**Table 1:** CDST active ingredients

A focus on enhancing the involvement of veterans in treatment processes, particularly related to treatment decision-making.	
Skills-based intervention strategies, including role-plays, worksheets, and in-session and out-of-session practice.	
Specific skills of assertiveness, problem-solving, goal planning, and managing conflict and disagreements.	
Psychoeducation on relevant topics.	
Active engagement by participants to identify goals and challenges in their current treatment processes.	
An empowerment-focused therapeutic approach.	

Following initial development and refinement based on feedback from people with SMI and providers who work with those with SMI, the manual was piloted in a civilian sample (*N* = 21) in the Midwest [34]. The pilot study results indicated that CDST is feasible, including high (>90%) average participant satisfaction, high (~89%) average attendance, high (~90%) therapist fidelity, and low (~11%) attrition during the treatment itself. CDST pilot participants experienced significant improvements in targeted skills and sense of personal recovery and decreased clinical symptom severity.

### Rationale for adaptation

There are a number of features that make this intervention appropriate for veterans and the VA context. For example, the VA and particularly the Psychosocial Rehabilitation and Recovery Centers (PRRCs) that serve veterans with SMI frequently utilize skills groups, and increasing veteran-provider collaboration is a priority for veterans and VA administrators alike. While CDST is likely to be a good fit for veterans with SMI receiving services at the VA, it is also likely that CDST will require some adaptation for this new setting and population. For example, this might include the vignettes and role-plays, which were developed in tandem with the patients and providers in the original civilian service environment. Some of those examples may not be applicable or useful to the veterans receiving VA care, or there may be additional topics that need to be addressed in this population.

CDST is a manualized intervention that has, to date, not been used in VA or with VA populations. Manualized or structured interventions are frequently modified to increase relevance for different patient populations and to increase feasibility and fit for new settings [37, 38]. Adaptations are unavoidable and necessary to ensure the adoption, implementation, and sustained use of interventions [39]. However, modifications often occur by providers reactively and without clear documentation. There are risks associated with this kind of approach: (1) adaptations may dilute or even remove the components of an intervention that make it effective (i.e., its “active ingredients”) and (2) it may be difficult for other settings to consistently deliver the modified intervention without comprehensive documentation. In the best case, manualized interventions provide direction for intervention content and intervention delivery approach after being tested with different populations and different settings.

### Aims

**Fig. 2:**
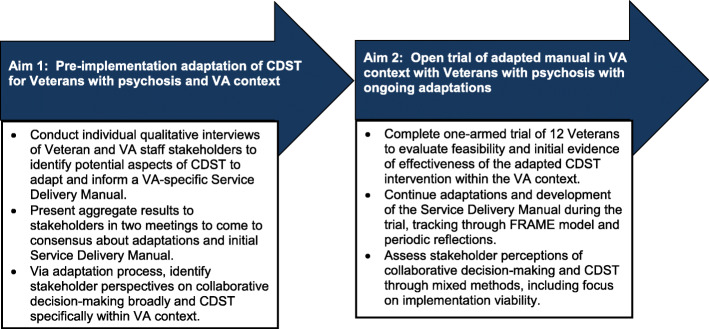
Visual of study aims

The proposed study has two interconnected aims (Fig. 2). Aim 1 is to identify how aspects of CDST or service delivery need to be adapted using a systematic approach to optimize its effectiveness, usability, and fit for veterans and VA implementation, and complete these pre-implementation adaptations. Aim 2 is to conduct a small, one-arm, open trial using the adapted manual to assess feasibility and preliminary effectiveness and identify and complete ongoing adaptations of CDST for veterans receiving care in PRRCs. These aims do not have associated hypotheses but rather four goals: (a) identify and complete any adaptations to the CDST Clinician Manual to increase the feasibility, fit, and relevance of CDST in PRRCs without compromising CDST’s “active ingredients”; (b) develop an “in progress” Service Delivery Manual to facilitate providers’ ability to deliver CDST in PRRC contexts; (c) identify stakeholder perspectives on collaborative decision-making broadly and CDST specifically; and (d) evaluate the feasibility and initial evidence of effectiveness of the adapted CDST intervention for veterans receiving care in PRRCs.

## Methods

### Setting and population

Veterans receiving services through a VA Psychosocial Rehabilitation and Recovery Center (PRRC) in Southern California will be recruited to participate. This PRRC serves veterans with psychotic disorders and GAF scores of 50 or below (e.g., significantly impaired daily functioning). The PRRC has an interdisciplinary treatment team currently including psychologists, psychiatrists, social workers, occupational therapists, and chaplains, and provides a range of individual and group services on an outpatient basis. The local PRRC’s service population is 86% men, 44% White veterans, 18% Black veterans, and 18% Hispanic veterans. Average age of PRRC veterans is 45 (SD =13), 11% identify as LGBTQ and 5% are homeless. In fiscal year 2018, 68% of veterans at this PRRC endorsed that one of their top goals is managing physical and mental health care, and 55% endorsed that social skills and interaction was a top goal, indicating that CDST could be a good fit for current veteran priorities.

### Aim 1: adaptation methods

An abbreviated version of the Intervention Mapping (IM Adapt) process will be used to increase the fit of the CDST Clinical Manual and CDST Service Delivery Manual to veterans and the context of the VA [40]. IM Adapt is a five step process that guides researchers and practitioners in selecting an evidence-based intervention (EBI), making decisions about whether and what to adapt, and executing the adaptation while guarding the EBI’s essential elements (those responsible for effectiveness). We will use steps 3 through 5 to guide our adaptation process. These steps include (3) plan adaptations based on fit assessments, (4) make adaptations and plans for implementation, and (5) plan for evaluation of the adapted Clinical Manual and CDST Service Delivery Manual. The CDST Clinician Manual includes the session-by-session contents, including the skills, worksheets, homework, role-plays, and prompts for clinicians. The Service Delivery Manual is focused on *how* the group is delivered, including ways that clinicians can change how they deliver the group while meeting fidelity expectations and ensuring that participants benefit. Although specific adaptations will be decided upon by this study’s stakeholder participants, in general, the adaptation to the Clinician Manual could include vignettes and role-play examples that better reflect the VA context, while the Service Delivery Manual could include recommendations for managing dynamics in the group, picking appropriate role-plays given group member needs, and approved service delivery variations (e.g., number of meetings per week). Adapting to changing contexts, like meeting virtually to accommodate COVID-19, could also be addressed in the Service Delivery Manual.

The existing CDST Clinical Manual from the pilot study will be modified during aim 1 based on findings from this project to increase fit for the new setting and population. The new CDST Service Delivery Manual will be developed during aim 1 and honed during aim 1 and aim 2 study activities. The goal of applying the abbreviated IM Adapt process in this study is to assess possible adaptation needs and meet those needs while maintaining fidelity to the intervention. IM Adapt protects the “active ingredients” of CDST and further adapts it, making it relevant to veterans and feasible in VA PRRCs. This will result in a Clinician Manual that is optimized to VA settings without loss of “active ingredients” and a Service Delivery Manual that enhances VA PRRC clinicians’ ability to deliver CDST effectively within their own service contexts. IM Adapt prioritizes integrating data with multiple stakeholder engagement to identify needs and values of stakeholders and translate them into practical results.

An Adaptation Resource Team (ART) will be formed including a minimum of 2 veterans with SMI currently receiving services in the PRRC, 2 clinicians who provide treatment in the PRRC, and 2 administrators whose duties include administration of the PRRC veterans will be enrolled via self-referral following announcements made during PRRC group therapy appointments and referrals from clinicians. Clinicians and administrators will be solicited directly. Informed consent will be obtained from each participant by a member of study staff. Potential participants with conservators will be approached for assent, and their conservators will complete informed consent; no person with a conservator will participate unless they assent.

#### Inclusion/exclusion criteria

Inclusion criteria for veterans in the ART are as follows: (1) currently receive services in the PRRC (i.e., seen in the clinic in the past month and/or completed a PRRC group during the past trimester); (2) have an SMI diagnosis (schizophrenia, schizoaffective disorder, delusional disorder, and major depressive disorder with psychotic features) per the electronic medical record; (3) are age 18 or above; (4) are fluent and literate in English.

Exclusion criteria for veterans in the ART are as follows: (1) having primary substance use or organic neurological disorder diagnosis; (2) are determined by PRRC and/or study staff to be at significant risk of exacerbation of symptoms, suicidal ideation, or other risk due to study participation; or (3) have a history and/or current risk of violence that PRRC and/or study staff determine to be too high risk to manage effectively at the PRRC’s outpatient clinic location. Risk of suicide and violence will be assessed via VA risk flag, impressions of the PRRC clinical team, and assessment by the study staff.

The following are inclusion criteria for PRRC clinicians and administrators in the ART: (1) currently engaged in the provision, direction, and/or administration of services in the PRRC.

#### Procedure

Each ART member will receive a copy of the Clinician Manual and a description of the purpose of the Service Delivery Manual. They will also receive a brief overview of the Clinician Manual in the event that they do not read the Clinician Manual prior to the interview appointment. They will be asked to review these materials and make notes of their perceptions, including components that are not relevant, may not resonate with the veteran population, or would not be feasible within the PRRC context. Approximately 2 weeks later, each participant will complete a semi-structured interview. These interviews will systematically work through the CDST content and service delivery aspects of the intervention. Interviews will be approximately 30–60 min, audio-recorded, and professionally transcribed*.* Additionally, study staff will contact all PRRC-associated staff to participate in these interviews if desired. Significant efforts will be made to recruit non-ART providers who do not have buy-in to CDST given that members of the ART are likely to have more buy-in to collaborative decision-making and CDST.

Following the interviews, study staff will compile the results and present them to the ART members. Over the course of two group meetings, facilitated by the first author, the ART members will provide feedback to inform initial development of the Service Delivery Manual and revisions to the Clinician Manual, to be used by providers of CDST during the open trial. Stakeholders may opt to meet with study staff individually instead based on preference or scheduling needs. Following these meetings, study staff including E.T. and W.S. (the CDST developers) will discuss these recommendations in the context of CDST’s “active ingredients,” to ensure protection of these ingredients while responding to stakeholder feedback.

We will take a primarily neo-positivist conceptualization [41] to the semi-structured interviews and ART team meetings, meaning that we assume that, if the interview questions are constructed well and the interviewers build rapport while remaining neutral, the participants’ responses will accurately reflect their perspectives. However, we also need to disclose and account for the relationship between the study team and CDST (i.e., E.T. and W.S. developed CDST, and E.T. will be the primary interviewer), along with pre-existing relationships with providers and multiple meetings intended for all ART members. Therefore, drawing upon the more transparent approach from the romantic conceptualization [41] will allow for disclosure of interviewer and research positionality in the research while providing the interviewers the opportunity to explicitly describe the goal of the work: to understand how to improve collaborative decision-making for veterans for those who want it, not to promote CDST regardless of its fit or effectiveness.

### Aim 1: analysis plan

We will use a rapid qualitative analysis technique tailored for solution-focused health service research [42]. This approach is intended for studies with clear, action-oriented goals, a team approach to data collection, and the use of intentional sampling, making it an excellent fit for our study [43]. Analysis will occur in tandem with data collection, so that collection of new data can respond directly to current understandings of past data. Matrix analysis [44] will be used to organize data and understand variation between interviews to help inform potential adaptations and other recommendations and questions for the ART meetings. Following completion of the individual interviews, the study team will complete initial analysis, yielding a presentation of results including possible adaptations for the ART meetings. Presentation to the ART will provide an opportunity for member checking to ensure that analysis was accurate and not only help identify the ideas and perspectives brought up by the ART members individually but also ensure the solution is agreed upon by the ART. New topics may be introduced during these meetings which can be added to the analysis and to the list of adaptations. All adaptations will be analyzed and organized using the expanded Framework for Reporting Adaptations and Modifications (FRAME) model [45]. Following consensus agreement, the initial adapted VA CDST Clinician Manual and Service Delivery Manual will be finalized in preparation for the open trial.

### Aim 2: open trial methods

In order to have a final sample of 10 veterans, we will recruit 12 veterans (estimating 20% attrition). CDST will be delivered within the context of the PRRC’s service menu, i.e., its timing, location, and staffing will all be congruent with how other groups are delivered at the PRRC. Open trial clinicians will be psychology and social work interns and fellows at the PRRC. They will receive training in CDST and will be supervised and monitored for fidelity by the primary developer.

Recruitment and all other data collection will align with existing treatment scheduling processes at the PRRC. Eligible veterans will be connected to our study via referrals from PRRC staff and study advertisements placed in PRRC spaces. Veterans may also self-refer. The consent process will be identical to aim 1.

#### Inclusion/exclusion criteria

Inclusion and exclusion criteria for veterans in the open trial are identical to the criteria for the ART, with one addition: veterans in the open-label trial must agree to have sessions audiotaped in order to participate.

##### Open-label trial participant assessments and assessment strategy (see Table 2)

Outcomes are organized using the RE-AIM framework [46], which is intended to facilitate comprehensive evaluation of the impact of a program or intervention. This will allow for examination of adaptation, feasibility, and preliminary evidence of effectiveness associated outcomes within the overall context of implementation feasibility. Veterans will receive compensation for assessment completion but not for intervention attendance to minimize the potential impact of compensation on treatment engagement. Data collection will be protected according to VA mandate to ensure participant confidentiality.

**Table 2:** Measures organized by timing and RE-AIM framework

RE-AIM	Targeted domains and measures	Throughout trial period	Baseline	Midpoint	Post	Follow-up	Chart review
Reach	**Representativeness of open trial sample**						
	Comparison of demographic data of enrolled participants to (a) all screened participants and (b) most recent aggregate census data provided by clinic						SS
	Comparison of demographic data of open trial completers to non-completers						SS
Effectiveness	**Primary outcome: veteran-provider collaboration**						
	Shared Decision Making Coding System (SDM-CS)		VS	VS	VS	VS	
	**Secondary outcome: collaborative decision-making perceptions and preferences**						
	Shared Decision Making Scale for Mental Health (SDM-MH-9)		V		V	V	
	Collaborative Decision Making Approach Measure (CD-MAM)		V		V	V	
	Problem-Solving Decision-Making Questionnaire for Mental Health (PSDMQ-MH)		V		V	V	
	**Secondary outcome: treatment engagement and motivation**						
	Non-study-related treatment attendance						VS
	Service Engagement Scale (SES)		R	R	R	R	
	Situational Motivation Scale for Schizophrenia Research **(**SiMS-SR**)**		V		V	V	
	Interest in adjunctive rehabilitation approaches		V		V	V	
	**Secondary outcome: treatment satisfaction**						
	CSQ: treatment satisfaction		V		V	V	
	**Secondary outcome: treatment outcome**						
	Canadian Occupational Performance Measure (COPM**)**		VS		VS	VS	
	Goal Attainment Scaling **(**GAS**)**		R		R	R	
	Maryland Assessment of Recovery in Serious Mental Illness **(**MARS**)**		VS		VS	VS	
	Brief Psychiatric Rating Scale **(**BPRS**)**		R		R	R	
	Social Skills Performance Assessment **(**SSPA**)**		VS		VS	VS	
	Personal and Social Performance Scale **(**PSP**)**		R		R	R	
	**Proximal outcome: targeted knowledge and skills**						
	Targeted skill gain		C	C	C		
	Targeted knowledge gain		V		V	V	
	**Exploratory outcome: patient-initiated collaborative behavior**						
	Consumer-Created Opportunities for Active Involvement Coding System (CCOAI-CS)		VS		VS	VS	
	**Exploratory outcome: clinician engagement in collaboration**						
	Shared Decision Making Coding System (SDM-CS)		VS		VS	VS	
	Consumer-Created Opportunities for Active Involvement Coding System (CCOAI-CS)		VS		VS	VS	
	**Exploratory outcome: acute service use**						
	Average frequency of crisis service use						VS
	**Multiple outcome domains**						
	Qualitative interviews		V		V	V	
Adoption	**Setting and provider characteristics**						
	Descriptive data of clinical setting and clinicians	SS, C					
Implementation	**Implementation feasibility for veterans and PRRC context**						
	Qualitative interviews		C		V, C		
	**Fidelity**						
	Therapist fidelity ratings	SS					
	Therapist supervision	SS					
	**Veteran participation**						
	CDST session attendance	C					
	Homework completion	C					
Maintenance	**Clinician and veteran buy-in**						
	Qualitative interviews		C		V, C		

*V* veteran participating in CDST open trial completes, *R* recovery coach of veteran participating in open trial completes, *VS* rating of veteran participating in CDST open trial completed by study staff, *C* clinician providing CDST during open trial completes, *SS* study staff completes

##### Reach

Representativeness of the open trial sample will be assessed three ways: first, by comparing the demographic data of the enrolled participants to all screened participants to detect any differences between those enrolled and those screened out; second, by comparing enrolled participants to aggregate census data provided by the clinic; and third, by comparing open trial completers to non-completers.

##### Effectiveness

###### *Primary outcome: veteran-provider collaboration*

The primary outcome, veteran-provider collaboration, is defined as number of collaborative decision-making behaviors utilized by veterans during appointments with PRRC providers. Audio recordings of veteran appointments with PRRC providers will be analyzed using the Shared Decision-Making Coding System (SDM-CS) [47], which is a validated method of coding collaborative behaviors during treatment decision-making among patients with SMI and their providers. Each veteran will have at least two appointments audio-taped at each point of the study: baseline, midpoint, post-intervention, and follow-up. Only non-study appointments will be recorded, including appointments with psychiatrists, therapists, and recovery coaches. The total number veteran appointments scheduled at each time point will vary by veteran, but generally, veterans in this PRRC meet with their psychiatrist monthly or quarterly, therapist weekly or biweekly, and recovery coach monthly, so at least two appointments are likely to be available for recording per study period.

###### *Secondary outcome 1: collaborative decision-making perceptions and preferences*

Veteran perceptions of collaboration will be assessed using two self-report measures, the Shared Decision-Making Scale for Mental Health (SDM-MH-9) and the Collaborative Decision-Making Approach Measure (CD-MAM) [34, 48]. Veteran preference for collaboration will be assessed using a vignette-based self-report measure, the Problem-Solving Decision-Making Questionnaire for Mental Health (PSDMQ-MH) [34].

###### *Secondary outcome 2: treatment engagement and motivation*

Treatment engagement and motivation will be measured via attendance at all non-study-related PRRC appointments: the Service Engagement Scale (SES) [49], a 14-item clinician-rated measure using a 4-point Likert scale; the Situational Motivation Scale for Schizophrenia Research (SiMS-SR; personal communication, K-H. Choi, March 2017), a 16-item self-report measure; and veteran self-reported interest in adjunctive rehabilitation activities currently offered via the PRRC.

###### *Secondary outcome 3: treatment satisfaction*

Satisfaction with PRRC services (i.e., not CDST itself, but rather the complete service package) will be assessed using the Client Satisfaction Questionnaire (CSQ) [50], an 8-item self-report measure of client satisfaction.

###### *Secondary outcome 4: improved treatment outcomes*

Four primary areas of outcome will be assessed: rehabilitation goal attainment, sense of personal recovery, symptom severity, and social functioning. The Canadian Occupational Performance Measure (COPM) [51] and the Goal Attainment Scaling (GAS) [52, 53] will be used to assess goal attainment. The Maryland Assessment of Recovery in Serious Mental Illness (MARS) [54] will be used to assess sense of personal recovery. The Brief Psychiatric Rating Scale, 26-item version (BPRS) [55–57], will be used to assess symptom severity. Two measures, the Social Skills Performance Assessment (SSPA) [58] and the Personal and Social Performance Scale (PSP) [59] will be used to assess functioning.

#### Proximal outcomes

##### Targeted knowledge and skills

Veteran gains in knowledge and skills targeted by CDST will be measured by clinician ratings of targeted skills and veteran performance on a quiz of targeted knowledge.

#### Exploratory outcomes

##### Patient-initiated collaborative behavior

For appointments where no decision is made, the Consumer-Created Opportunities for Active Involvement Coding System (CCOAI-CS) will be used instead of the primary outcome measure (i.e., the SDM-CS) to measure collaborative skills used in non-decision-focused appointments. The CCOAI-CS [60] is a validated measure for adults with SMI that codes presence of patient-driven collaborative behaviors, including sharing opinions about treatment effectiveness, making requests, and reflecting on the therapeutic relationship.

##### Clinician attitudes and behavior

Changes in clinician behavior during treatment decision-making will be assessed via the SDM-CS as described above. We will also explore ability to use the CCOAI-CS to identify clinician responsivity to patient-driven collaboration. There is not yet a standardized assessment of clinician attitudes towards collaborative decision-making. Therefore, changes in clinician attitudes will be assessed during the qualitative interviews.

##### Acute service use

Emergency room (ER) visits, crisis calls, and stays at acute inpatient units will be assessed through review of CPRS records. Baseline rates will be assessed through frequency in the 12 months prior to the start of the study and then translated into a per month rating. Baseline frequency will be compared to frequency during the intervention (i.e., baseline through post-intervention assessment) and after intervention completion (after post-intervention assessment through follow-up).

##### Multiple domains

Veterans will complete semi-structured qualitative interviews at baseline, post-intervention, and follow-up. Baseline interviews will broadly focus on veterans’ perception of their overall experiences in mental health care, including treatment decision-making, and how their level of involvement in treatment decision-making may impact other aspects of treatment (e.g., engagement) and their health. Post-intervention and follow-up interviews will focus on veterans’ experience in CDST, any changes they have noticed in themselves during or following participating in the study, including in the outcomes of interest, and any changes they have made in how they approach treatment decision-making. However, given that these interviews will be semi-structured, specific topics will vary based on individual veterans and other factors as the study evolves.

##### Adoption

Setting and provider characteristics will be assessed via descriptive data of the clinical setting and the clinicians who provide CDST.

##### Implementation

Implementation feasibility will be assessed via semi-structured qualitative interviews with the PRRC clinicians who provided CDST. Clinician interviews will broadly focus on their perceptions on CDST as a whole as well as specific elements, its fit and applicability for veterans with SMI and the PRRC context, veteran benefit and engagement, and clinician and administrator interest and likelihood of adoption and needed adaptations. Additionally, veteran participants will have the option to do a second post-intervention interview to give specific feedback on elements of CDST. These interviews will focus on veterans’ feedback about specific elements of CDST including elements that might require adaptation, how well CDST applied to them and veterans generally, and perception of veteran benefit, interest, and engagement. Veteran participation in the open trial will be assessed via attendance in CDST session and homework completion. Additionally, fidelity will be assessed through trained study staff ratings of clinician fidelity to CDST combined with supervision. We will also continue documenting adaptations in a systematic way using FRAME [45].

##### Maintenance

This study will not monitor long-term maintenance of implementation of CDST; instead, the qualitative interviews of the clinicians and veterans will be used to assess buy-in to inform potential for implementation beyond the research period.

### Aim 2: analysis plan

#### Analysis of open trial qualitative interviews

The qualitative transcriptions will be analyzed using content analysis, which is a structured technique that derives themes from qualitative data [61, 62]. Content analysis will be conducted by interview and stakeholder type. Open trial veteran interviews will be analyzed by time point. This will allow to differentiate between perspectives and needs of varying stakeholders. Content analysis will be conducted using NVivo. A frequency analysis will determine words that are most frequently used. Highly used words will be evaluated in the context of the transcription to determine commonalities in usage. This will inform initial development of themes. After initial development of themes, the entire transcript will be evaluated to determine quotes that inform an existing or new theme. Themes may be modified, added, or removed during the analysis process as the transcription is better understood. Interviewers will keep thorough field notes of meetings with stakeholders in order to triangulate transcription data, deepen understanding of qualitative data, and ease interpretation.

#### Analysis of adaptation during the implementation

Adaptations occurring during the open trial will be tracked using the expanded FRAME model [45]. Additionally, at least six periodic reflections [63] will be completed during the study period, with study staff, CDST clinicians, and other PRRC staff to help document implementation phenomena, including adaptations, changes in context, and challenges.

#### Analysis of open trial quantitative data

These data will be assessed using descriptive statistics rather inferential statistics. Effect sizes will be calculated for each measure by determining change in scores between baseline and post-intervention, and between baseline and 3-month follow-up. Cohen’s *d* effect size calculation and 95% confidence intervals [64] will be used for this purpose. In general, the purpose of these calculations is to verify that the direction of the effects is as expected, i.e., that CDST is associated with improvements in targeted areas. Although we will report the size of the effects and anticipate that some may be of medium to large size, given the large effects found in the civilian pilot [34], we are simultaneously aware that due to the small sample size, the confidence intervals may be large, requiring a larger sample for a precise estimate of effect size. Therefore, the primary goal will be to characterize direction of the effects rather than magnitude. Additionally, given the small sample size, we will also explore outliers using Cook’s distance. If we identify one or more outliers for a given effect, we will attempt to identify potential causes for the outlier effect by examining the outlier participants’ data in full context, including their qualitative data. This examination may also help us understand variables to consider for stratification during future larger trials.

## Discussion

Pursuit of recovery-oriented care is inextricably linked with evidence-based practice in mental health care. The VA recognizes the importance of both, and has made considerable strides to deliver recovery-oriented, evidence-based care to veterans with SMI. Increased collaborative decision-making is one area that remains to be improved, and there are, at present, no existing interventions tailored specifically for this population. CDST is an excellent candidate for this gap due to its development for civilians with SMI and its recovery-oriented focus that prioritizes patient empowerment and the development of generalizable knowledge and skills that patients can choose to use in any decision-making context with any treatment team member. This protocol will identify adaptations needed for CDST to appropriately fit the VA PRRC context and the needs and priorities of the veteran population, and conduct the first open trial of CDST (or indeed, any collaborative decision-making intervention) among veterans with SMI. This protocol will integrate FRAME and periodic reflections to track adaptations and uses RE-AIM to organize outcome domains. Mixed methods will be used to assess the open trial results. Results will be disseminated broadly, including to VA clinicians, veterans, and other stakeholders, as well as to scientific journals and the public.

There are methodological limitations to note. The adaptation process will focus specifically on the local VA PRRC and the veterans served by that PRRC. This VA and its service population may differ from other VAs in meaningful ways, and other VAs and PRRCs may find that the adaptations and Service Delivery Manual completed during this study does not fully capture all of their adaptation needs. Future studies that integrate other sites may be necessary to ensure that all adaptation needs are covered prior to all-VA implementation of the resulting CDST manual. This protocol describes a small open trial with no comparison group. While the study will yield useful information about feasibility and initial evidence of effectiveness, a larger trial with a comparison group will be necessary to fully understand the impact of CDST. These limitations are expected given the scope of this stage of the research. Other changes could potentially be made to the protocol as the study unfolds. For example, as of submission of this paper, data collection is set to begin in summer 2020, during the COVID-19 pandemic. The first stage of the study, the ART, will be completely virtual. The second stage may also need to be translated to virtual data collection depending on the progression of the pandemic to protect the safety of the participants and staff. The research staff will continue to follow both VA Research and VA PRRC guidelines. Key questions for the ART will include if and how to adapt CDST for video/phone groups instead of in-person. Other groups in this clinic have been delivered this way, so the ART’s perceptions of ways to do this successfully will be prioritized in decisions about how to proceed with the open trial. Any changes made will be documented and reported to relevant parties potentially including ethics boards, trial registries, funding groups, and journals.

Therefore, this study is novel and innovative, with the potential to have significant impact on veterans with SMI receiving care in PRRCs. Once adapted, CDST will fill an existing gap in care. The results of this study will inform feasibility and potential to improve collaborative decision-making and associated targeted outcomes including treatment engagement, satisfaction, and rehabilitation goal attainment for veterans with SMI. Additionally, these data will also increase the field’s understanding of perceptions of collaborative decision-making among veterans with SMI and VA clinicians, and result in a practical Service Delivery Manual that may be used by clinicians and administrators in VA PRRCs. Future studies may build upon the findings of this study in multiple ways, including a larger trial of CDST. Additionally, understanding typical decision-making processes, perceptions of those processes, and their relationships to key outcomes in VA PRRCs may elucidate other potential intervention pathways to improve collaborative decision-making specifically, and quality of care broadly, in these settings. Continued pursuit of interventions and system processes with high implementation feasibility that facilitate collaborative decision-making is key to improving quality of life and quality of care for veterans with SMI.

## Data Availability

Not applicable; data resulting from the planned study will be made available as described in the clinicaltrials.gov registration.

## Abbreviations

ART: Adaptation Resource Team
BPRS: Brief Psychiatric Rating Scale
CCOAI-CS: Consumer-Created Opportunities for Active Involvement Coding System
CDMAM: Collaborative Decision-Making Approach Measure
CDST: Collaborative Decision Skills Training
COPM: Canadian Occupational Performance Measure
CSQ: Client Satisfaction Questionnaire
EBI: Evidence-based intervention
ER: Emergency room
FRAME: Framework for Reporting Adaptations and Modifications
GAS: Goal Attainment Scaling
IM Adapt: Intervention Mapping
LGBTQ: Lesbian, gay, bisexual, trans, queer
MARS: Maryland Assessment of Recovery in Serious Mental Illness
NEPEC: Northeast Program Evaluation Center
PRRC: Psychosocial Rehabilitation and Recovery Center
PSDMQ-MH: Problem-Solving Decision-Making Questionnaire for Mental Health
PSP: Personal and Social Performance Scale
RE-AIM: Reach, Efficacy, Adoption, Implementation, Maintenance
SDM-CS: Shared Decision-Making Coding System
SDM-MH: Shared Decision-Making Scale for Mental Health
SES: Service Engagement Scale
SiMS-SR: Situational Motivation Scale for Schizophrenia Research
SMI: Serious mental illness
SSPA: Social skills performance assessment
VA: Veterans Affairs
